# Cardiovascular health and mortality in Cushing’s disease

**DOI:** 10.1007/s11102-022-01258-4

**Published:** 2022-07-22

**Authors:** Amy Coulden, Ross Hamblin, John Wass, Niki Karavitaki

**Affiliations:** 1 Institute of Metabolism and Systems Research, College of Medical and Dental Sciences, University of Birmingham, Birmingham, UK; 2 Centre for Endocrinology, Diabetes and Metabolism, Birmingham Health Partners, Birmingham, UK; 3 Department of Endocrinology, Queen Elizabeth Hospital, University Hospitals Birmingham NHS Foundation Trust, Birmingham, UK; 4 Department of Endocrinology, Oxford Centre for Diabetes, Endocrinology and Metabolism, Oxford University Hospitals NHS Foundation Trust, Oxford, UK

**Keywords:** Cushing’s Disease, Hypercortisolemia, Cardiovascular Health, Mortality

## Abstract

Exposure to cortisol excess in Cushing’s disease (CD) results in increased cardiovascular morbidity and reduces survival, with cardiovascular disease being a leading cause of death. At diagnosis, a significant number of patients have adverse cardiovascular profiles (e.g., obesity, diabetes or impaired glucose tolerance, dyslipidemia, hypertension, cardiac abnormalities and vascular disease). Remission of hypercortisolemia reduces but does not completely eliminate the cardiovascular complications; hazard ratios for myocardial infarction and stroke are high during long-term monitoring, highlighting the long-lasting effects of hypercortisolism and the importance of the timely diagnosis and successful management of this condition. Data on mortality of patients in remission are not consistent but in a multicenter study, an increased all-cause and circulatory mortality in patients with CD in remission for at least 10 years has been demonstrated. Cardiovascular morbidity requires particular focus and effective management during the care of patients with CD, from their presentation until long-term follow up.

## Introduction

Glucocorticoid excess causes several abnormalities leading to increased cardiovascular morbidity and mortality, which often do not normalize, even after achievement of biochemical remission [1]. In this review, we will focus on the impact of Cushing’s disease (CD) on the cardiovascular health and on the mortality of patients with this condition.

## Metabolic manifestations

### Obesity

Weight gain is frequently reported at diagnosis of CD, with obesity affecting 43–68% of patients [2]. There is also central fat distribution, with higher total, visceral and trunk subcutaneous adipose tissue than in matched controls [3]. Following remission, a decline in total, visceral and subcutaneous fat, as well as in weight and waist circumference has been observed [4]. However, by 5 years post-remission, 73% of patients remain overweight or obese [5] (Table 1).

**Table 1:** Prevalence of Cushing’s disease related co-morbidities at diagnosis, at intervals between 6 weeks and 12 months after remission, and at last assessment

Morbidity		Parameter	At diagnosis/active disease	6 weeks to 12 months after remission	At last assessment
BMI	Hassan-Smith et al., 2012 [2]	BMI (kg/m2) [IQR]	30 [27–35]	29 [27–34]^a^*	27 [23–32]^b^*
	Geer et al., 2012 [4]	BMI (kg/m2) [± SD]	32.1 [± 5.6]	28.9 [± 6]^c^	NA
		WC (cm) [± SD]	103 [10.6]	95.6 [15.1]^d^	NA
IGT	Giordano et al*.,* 2011 [6]	Prevalence (%) [n]	14 [2/14]	14 [2/14]	NA
	Faggiano et al*.,* 2003 [7]	Prevalence (%) [n]	64 [16/25]	NA	NA
	Ntali et al*.,* 2013 [8]	Prevalence (%) [n] IGT or diabetes mellitus	22 [67/311]	NA	17 [53/311]^e^**
Diabetes mellitus	Hassan-Smith et al., 2012 [2]	Prevalence (%) [n]	31 [22/72]	NA	NA
	Giordano et al*.,* 2011 [6]	Prevalence (%) [n]	36 [5/14]	14 [2/14]	NA
	Faggiano et al*.,* 2003 [7]	Prevalence (%) [n]	20 [5/25]	12 [3/25]	NA
		FBG (mmol/L) [± SD]	5.6 [± 0.4]^f^	5.0 [± 0.4]	NA
		Blood glucose 2 h after OGTT (mmol/L) [± SD]	9.4 [± 0.6]^g, h^	8.5 [± 0.7]	NA
Dyslipidemia	Hassan-Smith et al., 2012 [2]	Prevalence (%) [n]	12 [9/72]	NA	NA
	Giordano et al*.,* 2011 [6]	Prevalence (%) [n]	64 [9/14]	50 [7/14]	NA
		Total cholesterol (mmol/L) [± SD]	5.7 [± 0.3]	5.4 [± 0.3]	NA
		LDL cholesterol (mmol/L) [± SD]	3.4 [± 0.3]	3.4 [0.2]	NA
		TG (mmol/L) [± SD]	1.8 [± 0.3]	1.9 [± 0.2]	NA
	Faggiano et al*.,* 2003 [7]	Total cholesterol (mmol/L) [± SD]	6.20 [± 0.65]^f^	5.5 [± 0.55]	NA
		LDL cholesterol (mmol/L) [± SD]	4.35 [± 0.6]^h,i^	3.75[± 0.5]^f^	NA
		Total: HDL cholesterol [± SD]	6.10 [± 0.6]^g, h^	5.1 [± 0.55]^h^	NA
Hypertension	Hassan-Smith et al., 2012 [2]	Prevalence (%) [n]	78 [56/72]	NA	NA
		SBP (mmHg) [IQR]	150 [130–162]	124 [110–150]^j^*	130 [116–140]^j^*
		DBP (mmHg) [IQR]	90 [80–100]	80 [76–90]^j^*	82 [76–86]^j^*
	Giordano et al*.,* 2011 [6]	Prevalence (%) [n]	64 [9/14]	50 [7/14]	NA
	Faggiano et al*.,* 2003 [7]	Prevalence (%) [n]	72 [18/25]	40 [10/25]	NA
		SBP (mmHg) [± SD]	141 [± 7.45]^h,i^	125 [± 3.9]	NA
		DBP (mmHg) [± SD]	99 [± 2.65]^g,h,i^	86.5 [± 2.45]^h^	NA
	Ntali et al., 2013 [8]	Prevalence (%) [n]	49 [151/311]	NA	38 [117/311]^k^**

*BMI* Body mass index, *WC* waist circumference, *IQR* Interquartile range, *SD* Standard deviation, *IGT* Impaired glucose tolerance, *FBG* Fasting blood glucose, *OGTT* Oral glucose tolerance test; *TG* Triglycerides, *SBP* Systolic blood pressure, *DBP* Diastolic blood pressure, *NA* Not available

^a^*p* = 0.006 *vs.* at diagnosis, ^b^*p* = 0.0004 *vs*. at diagnosis, ^c^*p* < 0.01 *vs.* pre-operative values (active disease), ^d^*p* < 0.05 *vs.* pre-operative values (active disease), ^e^*p* = 0.02 *vs.* at diagnosis, ^f^*p* < 0.05 *vs.* sex- and aged-matched controls, ^g^*p* < 0.05 *vs.* BMI-matched controls, ^h^*p* < 0.01 *vs.* age- and sex-matched controls, ^i^*p* < 0.05 *vs.* remission, ^j^*p* < 0.0001 *vs.* at diagnosis, ^k^*p* = 0.001 *vs* at diagnosis

*Remission at 6 weeks reported in 83% of patients, remission at final assessment reported in 72% of patients. ** Remission at final assessment reported in 77% of patients

### Impaired glucose metabolism

CD can lead to impaired glucose tolerance and diabetes mellitus [2, 6–8]. Protracted hypercortisolemia impairs β-cell function and reduces insulin sensitivity in the liver, skeletal muscle and adipose tissue, resulting in gluconeogenesis [1]. After disease remission, abnormalities in glucose metabolism improve but do not completely resolve [6, 7]. Ntali et al*.* showed a significant reduction in the frequency of impaired glucose tolerance/diabetes mellitus type 2 from diagnosis (67/311, 22%) to last assessment (53/311, 17%), in a cohort with remission achieved in 77% of patients [8] (Table 1).

### Dyslipidemia

Dyslipidemia in CD is characterized by high triglycerides and total cholesterol levels with variable changes in HDL levels [9], and is frequently present in active disease [2, 6, 7]. Glucocorticoid excess leads to increased lipolysis and free fatty acid production and subsequent hepatic steatosis [9]. Dyslipidemia improves but does not normalize after remission [6, 7]; Faggiano et al*.* found a significant reduction in LDL cholesterol between active disease and at 1 year remission but not in total cholesterol [7] (Table 1).

## Cardiovascular manifestations

### Hypertension

In active CD, hypertension is highly prevalent (49–78%) and is associated with the duration and severity of glucocorticoid excess [2, 6, 8, 10] (Table 1). The pathogenesis is multifactorial; increased mineralocorticoid action, increased production of vasoconstrictors, such as endothelin-1 and enhanced reactivity of the cardiovascular system to vasoconstrictors, modulation of renin–angiotensin–aldosterone-system activity, inhibition of vasodilator release (e.g., nitric oxide, prostaglandins E2 and I2) and up-regulation of the sympathetic nervous system [10]. Despite remission, in two series, hypertension persisted in 50% (at diagnosis 64%) and 40% (at diagnosis 72%) of patients [6, 7], likely due to irreversible structural cardiovascular changes and vascular remodeling.

### Cardiac

Patients with active CD have left ventricular (LV) hypertrophy and concentric remodeling [11]. Systolic and diastolic dysfunction result from significant reduction in LV, right ventricular (RV) and left atrial (LA) ejection fractions [12]. Patients can rarely present with dilated cardiomyopathy or overt cardiac failure [12]. Pathogenesis includes enhanced response to angiotensin II, mineralocorticoid receptor activation and increased myocardial fibrosis [1]. At six-months post-remission, LV, RV and LA ejection fractions improve with a reduction in LV mass [12]. However, cardiac remodeling and relative wall thickness are still higher in patients in remission compared to controls [11]. Of note, hypokalemia can be present due to stimulation of the mineralocorticoid receptor by high cortisol levels predisposing to fatal arrhythmias [1].

### Vascular

Vascular aberrations complicate active CD with increased intimal media thickness (IMT) of major arteries [7]. There is also increase in the formation of atherosclerotic plaques [5] and arterial thrombosis [1]. Dekkers et al*.* found raised risk of ischemic heart disease (IHD) (HR 3.6, 95% CI 2.2–5.9) and stroke (HR 2.1, 1.2–3.6) in CD patients compared to controls [13]. Pathogenesis is multifactorial; low grade inflammation, microvascular endothelial dysfunction and hypercoagulability all contribute [1]. After remission, there is a relative resolution in vascular abnormalities (reduced IMT and increased diameter of the carotid artery) [7]. However, at 5 years post -remission, coronary artery plaques persisted in 27% of patients *vs.* 3% of controls [5]. The risk of stroke and IHD remains above that of the general population even in long-term remission [1].

## Mortality

The all-cause and cardiovascular-related mortality in patients with CD have been widely studied [2, 8, 13–15]. Patients with persistent disease have an undoubtedly high standard mortality ratio (SMR) [15]. Mortality data on patients in remission are not consistent; attributed to differences between studies (variability in criteria defining remission, in duration of cortisol excess, in recurrence rates, follow-up periods and therapeutic approaches) [15]. Notably, Clayton et al*.* reported increased all-cause mortality in patients with CD who had been in remission for at least 10 years at the time of entering the study (SMR 1.61, 95% CI 1.23–2.12); this group had median follow-up of 11.8 years from study entry and had remained in remission during the observation period [14].

Cardiovascular disease is a leading cause of death in patients with CD [1, 8]. Ragnarsson et al*.* in a series of 502 patients with CD (83% in remission) over a median follow up of 13 years, reported that cardiovascular disease was the commonest cause of death (SMR 3.3, 95% CI 2.6–4.3); IHD SMR 3.6 (95% CI 2.5–5.1) and stroke SMR 3.0 (95% CI 1.4–5.7). SMR related to circulatory disease of patients with active CD was even higher (9.5, 95% CI 4.15–17), and remained elevated even after remission (2.5, 95% CI 1.8–3.4) [16]. In the Clayton et al*.* series, circulatory SMR was 2.72 (95% CI 1.88–3.95) [14].

Predictors of elevated mortality remain unclear, as data on the impact of age at CD diagnosis, gender or pituitary radiotherapy are discordant [15].

## Conclusions

CD is associated with compromised cardiovascular health; this is attributed to several metabolic and cardiovascular morbidities, which may persist even after apparent biochemical remission, highlighting the long-lasting effects of hypercortisolism and the importance of timely diagnosis and successful management of this condition. Mortality in active CD is elevated and a number (but not all) studies suggest that it is also adversely affected in patients in remission. Cardiovascular disease is a leading etiology of death. A thorough assessment of CD-related cardio-metabolic changes, pre-existing co-morbidities, as well as family history and lifestyle factors must be undertaken at diagnosis of CD. Furthermore, recurrent screening of cardiovascular risk factors, even after biochemical remission, is mandatory [15], and modifiable risk factors and co-morbidities must be meticulously treated as per recent consensus [17].
